# Molecular Epidemiology of Cryptosporidiosis on Lamb and Goat Kid Farms in Gran Canaria, Canary Islands (Spain)

**DOI:** 10.3390/microorganisms13030644

**Published:** 2025-03-12

**Authors:** María Cristina Del Río, Sergio Martín, Joaquín Quílez, José Manuel Molina, Otilia Ferrer, José Adrián Molina, Adrián Melián, Antonio Ruiz

**Affiliations:** 1 Department of Animal Pathology, Faculty of Veterinary Sciences, University of Las Palmas de Gran Canaria (ULPGC), 35413 Arucas, Spain; mariacristina.delrio@ulpgc.es (M.C.D.R.);; 2 Department of Animal Pathology, Faculty of Veterinary Sciences, University of Zaragoza, 50013 Zaragoza, Spain; 3 Instituto Universitario de Investigaciones Biomédicas y Sanitarias (IUIBS), University of Las Palmas de Gran Canaria (ULPGC), 35016 Las Palmas, Spain

**Keywords:** *Cryptosporidium*, sheep, goat, zoonosis, questionnaire, risk factors

## Abstract

The aim of this study was to analyse and characterise *Cryptosporidium* spp. in sheep and goats in Gran Canaria (Spain) and to identify the risks and economic factors related to the disease. During sampling, a semi-structured survey was conducted with farmers, and faecal samples were collected from lambs, goat kids, sheep, and adult goats from a total of 30 farms. Adult samples were examined microscopically for the presence of *Cryptosporidium* spp. oocysts, with only three positive samples being found in sheep and one in goats. The PCR of the *SSU rRNA* gene was performed on all juvenile and adult samples, and positive samples from lambs (8.3%), sheep (6.9%), goat kids (23.3%), and goats (2.5%) were subjected to sequencing, detecting three of the most important species in small ruminants: *C. parvum*, *C. xiaoi*, and *C. ubiquitum*. By sequencing the *GP60* PCR products, two subtypes of *C. parvum* belonging to the IId family were identified, IIdA16G1 and IIdA23G1, with the latter being the most frequent. Although the prevalence of the disease was not very high, the zoonotic potential of *C. parvum* and the limited awareness of the parasite among farmers make surveillance and health education focused on the control of this member of Apicomplexa necessary.

## 1. Introduction

*Cryptosporidium* spp. are protozoan parasites of the phylum Apicomplexa that cause diarrhoeal cryptosporidiosis, a disease affecting both humans and livestock. This condition is associated with high mortality rates in young and immunosuppressed individuals. It has long been classified as a coccidian due to its life cycle similarities with such parasites, but *Cryptosporidium* is now believed to bear a closer molecular and biological resemblance to gregarine parasites. Transmission typically occurs via the faecal–oral route, often through contaminated food or water. Infection leads to malabsorptive diarrhoea by destroying the epithelium of the small intestine, causing villous atrophy and crypt hyperplasia [1,2,3].

This Apicomplexa parasite is known to infect more than 150 mammal species, including humans, fish, amphibians, reptiles, and birds. To date, more than 40 host-specific species have been identified. In humans, 21 different species have been documented, although most infections are caused by *C. hominis* and *C. parvum*. Notably, *C. parvum* has also been reported in a wide range of animal hosts [1,3,4,5,6,7,8,9,10].

In recent years, there has been a notable increase in cryptosporidiosis outbreaks among humans, often linked to contaminated water and livestock as sources of infection [11,12,13].

In the livestock sector, *Cryptosporidium* spp. have been reported across all types of husbandry systems, causing significant economic losses due to increased veterinary costs, reduced weight gain, and high mortality rates [8,14]. Furthermore, the control and elimination of this protozoan from infected farms poses a substantial challenge, not only because the infectious dose is low [15,16,17,18] but also due to the highly resistant outer shells of oocysts. This shell enables the parasite to survive for extended periods in the environment, withstanding temperature extremes (−22 °C to 60 °C) and most farm disinfectants [18,19]. These control challenges result in most infections occurring in neonates, which are exposed to the parasite shortly after birth. Infected neonates begin shedding large numbers of oocysts at two weeks of age in calves and between one and four weeks of age in lambs and goat kids [20,21,22,23,24]. While the transmission of *Cryptosporidium* spp. in small ruminants, such as lambs and goat kids, has received less attention compared to calves, research indicates that adult sheep and goats with subclinical infections can serve as a source of infection for young ruminants. This could be particularly evident during the peripartum period, where increased oocyst excretion is observed [25,26].

The use of the small subunit ribosomal RNA (*SSU rRNA*) gene has enabled the identification of different *Cryptosporidium* species in most countries worldwide, significantly enhancing our understanding of its epidemiology, particularly the means of disease transmission [27,28]. The most common *Cryptosporidium* species in small ruminants are *C. parvum*, *C. ubiquitum*, and *C. xiaoi* [29,30,31]. Other species found more sporadically in sheep are *C. andersoni*, *C. scrofarum*, *C. bovis*, *C. ryanae*, *C. hominis*, *C. fayeri*, and *C. suis*, whereas *C. hominis*, *C. baileyi*, and *C. andersoni* have been reported in goats [6,29,32]. Despite these findings, research on this parasite in small ruminants remains limited, and many aspects are still unclear, including the public health implications of species such as *C. xiaoi* or the potential relationship between the species and host age, as observed in calves [24,33,34]. Notably, some studies suggest that *C. ubiquitum* is more common in older animals, while *C. parvum* and *C. xiaoi* are more frequently found in lambs and goat kids younger than one month old [25,35,36].

Molecular subtyping tools have significantly advanced the study of *Cryptosporidium* transmission between humans and ruminants. One of the most widely used subtyping methods is the DNA sequence analysis of the 60 kDa glycoprotein, also known as *GP60* or *GP40*/*15*. In *C. parvum*, 14 subtype families (IIa to IIo) have been identified. Among these, subtypes within families IIb, IIc, and IIe have been exclusively detected in humans, while those in families IIa and IId are found in both humans and ruminants. Subtypes belonging to the IIa family are more commonly detected in calves, whereas subtypes within the IId family are more frequently identified in lambs and goat kids [6,7,36,37,38,39,40].

In the Canary Islands, sheep and goats have a greater impact on production than cattle, with a total census in 2023 of 200,054 goats and 40,399 sheep compared to 20,629 cattle [41]. In Mainland Spain and other arid and semi-arid areas around the world, small ruminant production is also of considerable economic and often sociocultural importance [42,43]. However, although *Cryptosporidium* has been detected in small ruminants in several regions of Mainland Spain [33,36,44,45], in the Canary Islands, only one study has analysed the incidence of this parasite in cattle [46], with no information available to date on its occurrence in small ruminants.

Cryptosporidiosis not only impacts sheep and goat farming but also poses a public health risk due to its zoonotic potential. As a result, it is crucial to develop and implement effective diagnostic and control measures. The main objective of this study was to determine the frequency of *Cryptosporidium* infection in goat and sheep farms across various municipalities on the island of Gran Canaria. The study also aimed to conduct the molecular characterisation of isolates and analyse the risk factors associated with cryptosporidiosis through farmer surveys.

## 2. Materials and Methods

### 2.1. Ethical Statement

All animal procedures were carried out in strict accordance with national ethics, the current European legislation on animal welfare (ART13TFEU), and protocols approved by the institutional review board (OEBA-ULPGC-37/2024).

### 2.2. Farms, Animals, and Sampling

**Figure 1:**
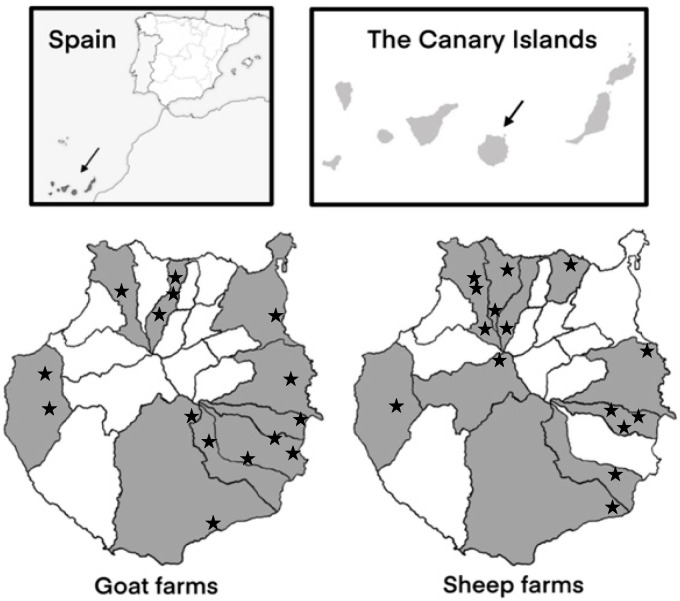
Geographical distribution of the 15 sheep farms and 15 goat farms sampled in the different municipalities of Gran Canaria (Spain).

The study was conducted on 15 sheep farms and 15 goat farms located in ten and nine municipalities of Gran Canaria (Canary Islands, Spain), respectively (Figure 1). Farms were selected based on variations in their size, production systems, management practices, and hygienic–sanitary measures. From each sheep farm, 5–8 faecal samples were collected from lambs aged 1–2 weeks and from 5–8 recently lambed ewes. Similarly, from goat farms, 8 samples were collected from goat kids within the same age range as the lambs, as well as 8 samples from recently lambed goats. The WinEpiscope software 2.0 (http://www.winepi.net/ (accessed on 13 December 2023)) was used to determine the number of animals sampled per farm.

Faecal samples were collected through digital stimulation directly from the rectum to avoid contamination. Samples were placed in sterile Eppendorf tubes, labelled, and assigned a faecal score based on their consistency: (1) normal faeces/no diarrhoea, (2) pasty faeces, (3) loose faeces, (4) liquid faeces, and (5) liquid faeces with blood or intestinal mucosa. The samples were transported under refrigeration to the Faculty of Veterinary Medicine at the University of Las Palmas de Gran Canaria (ULPGC), where they were stored at 8 °C until processing. In total, 410 individual faecal samples were collected and analysed throughout the study: 84 were from lambs, 86 from ewes, 120 from goat kids, and 120 from adult goats. Apart from specific microscopic and molecular analyses, no additional methods to detect other pathogens causing neonatal diarrhoea were addressed.

All experimental procedures followed institutional review board-approved protocols (OEBA-ULPGC-37/2024).

### 2.3. Microscopy Analysis

For adult animal samples, an ether sedimentation concentration technique followed by Kinyoun staining was used to enhance the detection of *Cryptosporidium* spp. oocysts by microscopic visualisation [46,47].

To evaluate the parasitic load in each sample, the estimation of the *Cryptosporidium* oocyst count (estimated oocyst count—EOC) was performed. Each sample was examined under a microscope (Panthera Series, Motic, Xiamen, China) at 1000× magnification for exactly 10 min. Oocyst counting was conducted in the areas of the smear with the highest staining quality, typically near the edges. As a general guideline, oocyst counts were performed across 50–80 randomly selected fields, unless the parasitic load was particularly high, in which case only 25 fields were examined. The infection intensity was categorised as follows: high (>25 oocysts), moderate (16–25 oocysts), mild (6–15 oocysts), minimal (1–5 oocysts), and no infection (0 oocysts) [46].

No concentration or staining technique was applied to neonatal samples due to the limited sample size. In this case, the entire sample was reserved for direct DNA extraction.

### 2.4. Molecular Analysis

#### 2.4.1. DNA Extraction

Following microscopic analysis, aliquots of faecal sediments from adult animal samples were stored at 4 °C for subsequent DNA extraction. DNA extraction from neonatal faecal samples and from adult sediment samples was performed using the E.Z.N.A.^®^ Stool DNA Kit—Omega Bio-Tek (Norcross, GA, USA), according to the manufacturer’s instructions. The extracted DNA was stored at −20 °C until polymerase chain reactions (PCRs) were conducted.

#### 2.4.2. PCR Primers and Conditions

Each sample was subjected to a nested PCR to amplify a fragment of the *SSU rRNA* gene and a single PCR to amplify a fragment of the 60 kDa glycoprotein gene (*GP60*). All primers used in this study have been previously described (Table 1). The PCR conditions and gel preparation were the same as those published in [46].

#### 2.4.3. DNA Sequence Analysis

Most samples selected for DNA sequencing exhibited a high amplicon intensity without non-specific bands. For *GP60* PCR-positive samples that displayed non-specific bands on the agarose gel, the specific band of interest was excised and purified using the E.Z.N.A.^®^ Gel Extraction Kit—Omega Bio-Tek (USA), following the manufacturer’s instructions. In total, 192 samples were submitted to Macrogen Europe Inc. (Madrid, Spain) for bidirectional Sanger sequencing. The sequences obtained from the sense and antisense strands were aligned using CLUSTAL W and subsequently edited with MEGA version 11.0.13 (https://megasoftware.net (accessed on 7 June 2024)). Consensus sequences were analysed using BLASTN searches against the NCBI databases (http://blast.ncbi.nlm.nih.gov/Blast.cgi (accessed on 30 June 2024)). Representative nucleotide sequences generated in this study were deposited in the GenBank database under the following accession numbers: PQ345453, PQ345455, PQ345467, PQ363713, and PQ363714.

#### 2.4.4. Phylogenetic Analysis

The sequences obtained from various genetic markers were compared against the GenBank database. Each *SSU rRNA* gene sequence was assigned to a specific *Cryptosporidium* species, while the *GP60* gene sequences were classified within *C. parvum* families and subtypes based on the TCA and TCG repeats in the trinucleotide repeat region and mutations in the non-repeat regions, as described in [49].

Phylogenetic analysis was conducted using the MEGA11.0.13 software. Neighbour-joining trees were constructed based on evolutionary distances calculated using the Kimura two-parameter model. To enhance the reliability of the trees, a bootstrap analysis was performed with 1000 replicates, with values below 50% being discarded. The neighbour-joining *SSU rRNA* and *GP60* trees were rooted using *Plasmodium cathemerium* (AY625607.1) and *C. parvum* IIcA5G3a (AY738195.1), respectively.

### 2.5. Questionnaire

After sample collection, farmers were surveyed to obtain information about the main risk factors and economic costs associated with ovine and caprine cryptosporidiosis in Gran Canaria. Veterinarians responsible for each farm provided technical data on treatments for diarrhoea and parasitic infections, vaccination practices, and other relevant information.

The questionnaire consisted of 23 questions addressing various topics: farm and farmer information (1/23), sheep and goat breeds (1/23), facilities and management practices (10/23), knowledge about cryptosporidiosis (2/23), treatments used for neonatal diarrhoea and their efficacy (3/23), clinical signs and outcomes of cryptosporidiosis (4/23), and direct or indirect costs associated with *Cryptosporidium* spp. infections (2/23).

Since the parasite load (EOC) based on the Kinyoun staining results could not be determined in neonatal samples due to limited faecal material, the molecular identification of the *SSU rRNA* marker was used as a standardised factor to assess associations with the questionnaire responses.

### 2.6. Statistical Analysis

Data on the faecal scores and PCR positivity for *SSU rRNA* were recorded in a Microsoft Excel^®^ table. A Z-test was conducted to compare the proportions of positive samples between young and adult animals, the host species, and the detection methods employed. The same test was used to compare the percentages of positivity among the different farms. Furthermore, a Spearman’s rank correlation test was used to analyse the relationship between PCR positivity for *SSU rRNA* and the faecal consistency in the animals. All statistical analyses were performed using the Sigmaplot 14.5 software, with statistical significance set at *p* < 0.05.

In order to address risk factors associated with caprine and ovine cryptosporidiosis, the surveys were digitised in Microsoft Excel^®^ for further analysis. Data on the *SSU rRNA* positivity and the number of infected animals per farm were compared against the questionnaire results. Dynamic tables were created to graphically represent potential correlations. The statistical analysis of these comparisons was performed using Fisher’s exact test, with the same software and significance threshold as described above.

## 3. Results

### 3.1. Parasitological Analysis and Faecal Score

Positive results using Kinyoun staining were detected in only 3.5% (3/86) of sheep samples from two farms and 0.8% (1/120) of goat samples. All positive cases were classified as having a minimal infection intensity.

**Figure 2:**
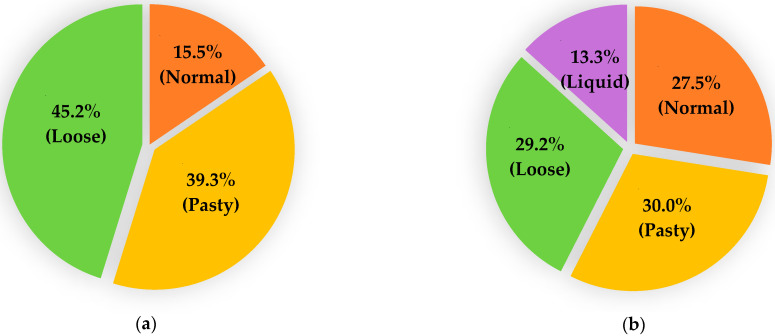
Comparison of faecal scores in lambs (**a**) and goat kids (**b**) sampled during the study.

No diarrhoea was observed in any adult animals, and all faecal samples were scored as “1”. However, a large variation in the faecal consistency was noted among young animals, ranging from normal (1) to loose (3) in lambs and from normal (1) to liquid (4) in goat kids (Figure 2). No significant differences in the faecal scores were observed between sheep and goats at either of the two age ranges assessed.

### 3.2. Molecular Analysis

#### 3.2.1. PCR Amplification of *SSU rRNA* and *GP60*

In general, Kinyoun staining detected fewer positives than the *SSU rRNA* gene PCR in both sheep and goat adults. Specifically, the *SSU rRNA* gene PCR identified *Cryptosporidium* in 20% of sheep farms, compared to 13.3% detected by microscopy. Among goats, 13.3% of farms tested positive using the *SSU rRNA* gene PCR, whereas 6.7% were positive under Kinyoun staining. At the individual level, the positivity rate in sheep increased from 3.5% (3/86) with Kinyoun staining to 6.9% (6/86) with the *SSU rRNA* gene PCR. In goats, the percentage increased from 0.8% (1/120) to 2.5% (3/120). However, no statistically significant differences were found at either the farm or individual level. Additionally, no samples from adult sheep or goats tested positive for the *GP60* marker.

The prevalence of *Cryptosporidium*-positive farms among lambs and goat kids was moderate to high, with detection rates of 33.3% and 60%, respectively, based on the *SSU rRNA* gene PCR. However, the number of infected individuals was relatively low, with positivity rates of 8.3% (7/84) in lambs and 23.3% (28/120) in goat kids for the *SSU rRNA* gene marker. Notably, only samples from goat kids were amplified for the *GP60* marker, with 5.8% (7/120) testing positive.

#### 3.2.2. Correlation Analysis Between *SSU rRNA* Gene Results and Faecal Scores

In lambs, 85.7% (6/7) of the *SSU rRNA*-positive animals also had diarrhoea, while, in goat kids, 89.3% (25/28) of the animals with a reduced faecal consistency tested positive for *SSU rRNA*. No adult *SSU rRNA*-positive animals exhibited diarrhoea.

The Spearman’s rank correlation analysis revealed a statistically significant relationship between the *SSU rRNA* gene PCR results and the type of diarrhoea observed in goat kids, with a correlation coefficient of −0.188 and a *p* value of 0.0402. This finding confirms the correlation between a higher faecal score classification and *SSU rRNA* gene PCR positivity. However, no such relationship was observed in lambs.

#### 3.2.3. Sequencing

The *Cryptosporidium* species, families, and subtypes identified by *SSU rRNA* and *GP60* sequencing, along with their corresponding frequencies, in the 30 sampled farms are shown in Table 2.

Three distinct sequences were detected using the *SSU rRNA* marker. The first sequence (accession number PQ345453) corresponded to *C. xiaoi* and was the most frequently identified across all age groups in both sheep and goats, particularly in goat kids; this species was present in six different farms. A comparison with sequences in GenBank revealed a 100% match with several previously published sequences. The second sequence (accession number PQ345455) matched *C. parvum* and was the second most prevalent species in this study. It was detected at a low frequency in lambs and adult sheep but was more common in goat kids. This *Cryptosporidium* species was found in five farms sampled, and this sequence was identical to more than 100 previously published *C. parvum* sequences in GenBank from multiple countries. The third sequence (accession number PQ345467) was identified as *C. ubiquitum*. It was detected in three lambs, four adult sheep, and two adult goats, but was not found in any of the tested faecal samples from goat kids. Similarly to the other two species, this sequence showed 100% homology with previously published sequences in GenBank.

All PCR-positive *GP60* gene products were obtained from isolates with *SSU rRNA* fragment sizes corresponding to *C. parvum*. *GP60* marker sequencing revealed that the two C. parvum sequences detected belonged to the IId family, with the subtypes differentiated based on the number of TCA repeats in the trinucleotide repeat region. The first *C. parvum* sequence (accession number PQ363713), belonging to subtype IIdA16G1, was detected in a single goat kid. A comparison with GenBank showed that only 11 previously published sequences exhibited 100% similarity. The second *C. parvum* sequence (accession number PQ363714), identified as subtype IIdA23G1, was detected in six goat kids from a single farm. Only one previously published sequence from Spain (PP333107.1) in GenBank showed 100% similarity. This sequence differed from the others due to an additional ‘TCA’ repeat.

#### 3.2.4. Phylogenetic Analysis

**Figure 3:**
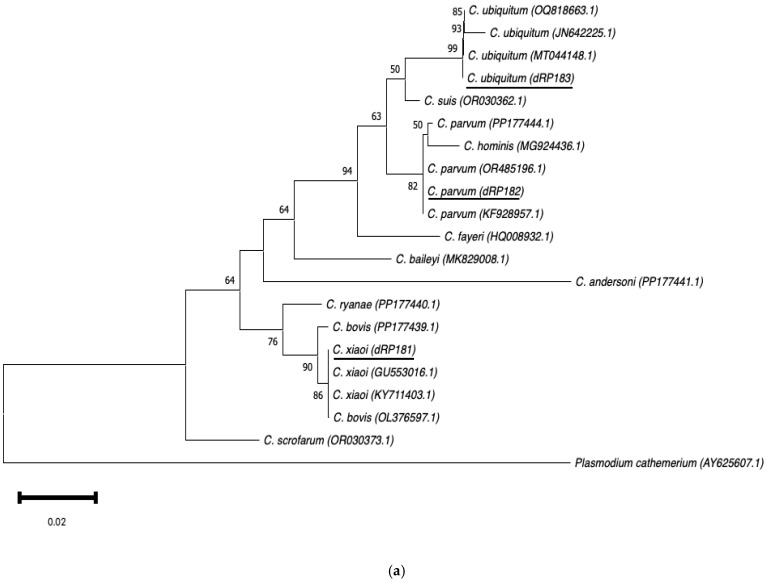

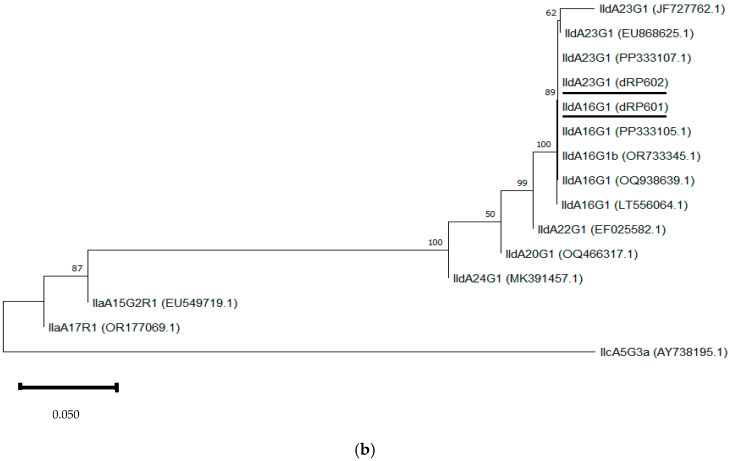
Phylogenetic analysis of the *SSU rRNA* (**a**) and *GP60* (**b**) loci using neighbour-joining trees based on the Kimura two-parameter model. Bootstrap values over 50% from 1000 pseudoreplicates are indicated at the left of the supported node. Scale bar indicates an evolutionary distance of 0.02 (**a**) and 0.050 (**b**) substitutions per site in the sequence.

Neighbour-joining trees were constructed using aligned *SSU rRNA* and *GP60* sequences obtained in this study, along with sequences downloaded from the GenBank database (Figure 3a,b). The *SSU rRNA* neighbour-joining tree showed that the sequences obtained clustered with those of the same *Cryptosporidium* species selected from GenBank. In the *GP60* neighbour-joining tree, three distinct groups were identified, corresponding to families IId, IIa, and IIc. The sequences IIdA16G1 (dRP601) and IIdA23G1 (dRP602) clustered together with other sequences of the same subtype from GenBank.

### 3.3. Questionnaire Analysis

#### 3.3.1. Farm Characteristics and Management Data

Among the fifteen sheep farms sampled, only one was classified as a familiar-sized farm (<30 animals), seven as small-sized (30–200 animals), six as medium-sized (200–600 animals), and one as a large-sized farm (>600 animals). Among the goat farms, five were classified as small-sized, four as medium-sized, and six as large-sized. All faecal samples from sheep and goats were collected from dairy farms. The majority of the sheep farms (12) had one lambing per year, while the remaining three had two lambing events per year. In the goat farms, a wider range of lambing frequencies was observed: seven had one lambing per year, five had two, two had three, and one had up to four lambing events per year.

In all sheep and goat farms, the animals were fed with a commercial mix. Additionally, hay and straw were used in nine sheep farms and six goat farms, while agricultural byproducts were provided in four sheep farms and two goat farms. Of the eleven sheep farms that practiced grazing, seven rotated the grazing sites. In contrast, only two of the seven goat farms that practiced grazing used different sites.

Regarding farm facilities, all goat farms had a milking parlour, whereas five sheep farms did not. Moreover, eight sheep farms and five goat farms had a cheese dairy. Additionally, eight sheep farms and nine goat farms had multiple pens to separate animals based on the production stage and age, while only five sheep farms and nine goat farms had designated areas for sick animals. Artificial lactation in specially designated areas was practiced in one sheep farm and four goat farms.

In terms of hygiene measures, only three goat farms removed manure daily, while three sheep farms and two goat farms removed it weekly. The remaining farms reported either never removing manure, as their animals grazed most of the time, or doing so at intervals of one month or longer. Most goat farms (13/15) cleaned and disinfected the cemented areas daily, whereas only four sheep farms followed the same routine. Parasitological analyses were conducted annually in one sheep farm and two goat farms.

Concerning immunization, four sheep farms and seven goat farms reported vaccinating against three to five diseases, while the remaining farms vaccinated against only one or two diseases. One sheep farm did not vaccinate against any diseases. Most sheep and goat farms did not implement preventive treatments against neonatal diarrhoea. The use of antiparasitic treatments was uncommon among the sampled farms, although toltrazuril (Baycox^®^, Bayer Animals Health, Monheim, Germany; Cenzuril^®^, Chanelle Pharmaceuticals Manufacturing, Loughrea, Ireland) was administered in one sheep farm and two goat farms, while albendazole (Albecorin^®^, CENAVISA, Tarragona, Spain) was used in one sheep farm. Additionally, most sheep and goat farms reported using antibiotics such as sulfamethoxazole/trimethoprim to treat neonatal diarrhoea, while three farms (sheep and goat) reported using no treatment at all.

#### 3.3.2. Parasitological Knowledge

Among the 15 sheep farms sampled, only one farmer reported being aware of cryptosporidiosis but considered it to be of minor importance. Regarding the goat farms, five farmers stated that they were familiar with the disease; however, three did not consider it significant, while two regarded it as of little importance. Two of the farmers who were aware of cryptosporidiosis—one sheep farmer and one goat farmer—were veterinarians. Both assessed the disease to be of little relevance to their herds.

#### 3.3.3. Economic Impact

Most herders reported that clinical signs associated with Cryptosporidium or coccidiosis were rare or non-existent, with only one goat farm experiencing a severe outbreak of diarrhoea. No sheep or goat farms invested in specific treatments for cryptosporidiosis. For other causes of neonatal diarrhoea, such as colibacillosis, four sheep farms and one goat farm spent less EUR 10 per year on treatment, while two goat farms spent between EUR 10 and EUR 50, one goat farm spent between EUR 100 and EUR 200, and one sheep farm spent more than EUR 200. The remaining farms did not apply any treatment for colibacillosis.

Sheep farmers reported spending less than two hours per year on controlling neonatal diarrhoea (12 farms), while two farms spent between two and four hours, and only one farm reported spending between 10 and 30 h. Among the goat farmers, most spent less than 2 h per year managing scours; however, four farms reported spending more than 30 h. On most farms, veterinarians reported spending less than 2 h or between two and four hours per year on neonatal diarrhoea management. Only two goat farms reported that veterinarians spent more than 30 h annually on diarrhoea control.

#### 3.3.4. Correlation Between Questionnaire and Parasitological Data

Due to the low number of infected animals detected by *SSU rRNA* analysis, establishing significant correlations between the PCR results and the evaluated risk factors was challenging. However, some notable correlations were identified. When analysing the management measures, a relationship was observed between the farm size and number of *Cryptosporidium*-positive farms both in kids and goats, with a higher percentage of positive individuals and farms in medium and large farms compared to small farms. Regarding goat kids, a higher percentage of positive herds was also detected on farms that used multiple pens for batch separation, practiced natural lactation, or had more than one kidding per year. Regarding lambs, a greater number of cryptosporidiosis-positive farms was observed in herds reared under intensive farming systems, while this association was less evident in goat kids. Regarding hygienic and sanitary measures, the only noteworthy correlation was a positive association between the consistency of the vaccination strategy and the presence of *Cryptosporidium* on the farm. No significant relationships were found between the *SSU rRNA* PCR data and the other evaluated parameters.

## 4. Discussion

This study investigated the occurrence of *Cryptosporidium* spp. in dairy ovine and caprine farms on the Spanish island of Gran Canaria (Canary Islands). Additionally, the genetic diversity of *Cryptosporidium* species was analysed, and *C. parvum* was characterised at the family and subtype levels. The findings indicate that, although the occurrence of cryptosporidiosis was low in both sheep and goats across the assessed age ranges, zoonotic species such as *C. parvum* and *C. ubiquitum* were present on farms in different municipalities of Gran Canaria. These results align with previous studies highlighting the importance of considering this parasite in the differential diagnosis of diarrhoea in small ruminants due to its public health significance and its potential to infect humans through contact with infected animals or contaminated environments and water sources [50,51,52].

The detection rate of *Cryptosporidium* in adult animals by microscopy was very low in both sheep (3.5%) and goats (0.8%), consistent with a study conducted in Turkey, where the prevalence in adult sheep did not exceed 2.4% [53]. However, other studies that included both adult and young animals have reported highly variable oocyst detection rates, such as 67.5% in sheep and 72.5% in goats from Mexico [54], 3.48% in goats from China [55], or 4.2% and 3.6% in sheep and goats from Kuwait, respectively [22]. Similarly, substantial variations in prevalence have been observed in studies focusing solely on young animals. For example, a high prevalence was reported in lambs (31.6–59%) and goat kids (62.7%) from Spain [45,56] and in lambs (42.1%) and goat kids (53.6%) from Serbia [57], whereas a much lower prevalence was recorded in lambs (1.8%) and goat kids (3.5%) from India [50]. These discrepancies among studies may be attributed to factors such as the age range, geographic conditions, sampling time, gender, sample size, examination method, management practices, hygiene conditions, and other variables [22,53,54].

Although PCR was more specific and sensitive than Kinyoun staining and identified a higher positivity rate in both adult and young animals, the prevalence of *Cryptosporidium* remained below 10%, except in goat kids, where nearly 25% of the sampled animals tested positive. This higher prevalence likely explains why a statistically significant relationship between the *SSU rRNA* gene results and faecal scores was found only in goat kids, with a greater number of positive animals detected as the faecal scores increased. These findings align with previous studies that have associated the presence of *Cryptosporidium* in sheep and goats with diarrhoea [29,45,58,59].

The *SSU rRNA* sequence analysis confirmed the presence of *Cryptosporidium* species in Gran Canaria. The following have been identified as the most important in sheep and goats by several authors: *C. parvum*, *C. xiaoi*, and *C. ubiquitum* [30,32,60]. The predominant species in lambs and sheep was *C. ubiquitum*, consistent with studies from Brazil [61] and France [62]. However, studies from China [63,64], the USA [65], Australia [66,67], and Scotland [68] have identified *C. xiaoi* as the most frequent species in sheep. Conversely, the predominant species in kids and goats was *C. xiaoi*, in agreement with studies from Greece [69], China [70], France [71], and Poland [72]. Nevertheless, *C. parvum* has been identified as the main species in sheep and goats in several European countries, including Spain [33,36,45], Italy [73], Belgium [74], and Romania [75], as well as in Asian or African countries such as Korea [76], Kuwait [22], and Israel [60].

In various countries, both the IIa and IId subtype families have been identified in large and small ruminants. However, several authors have reported that subtype IIa is more prevalent in calves, whereas IId is more common in lambs and goat kids [7,36,37,77]. In certain regions of Mainland Spain, subtypes IIaA13G1R1, IIaA14G2R1, IIaA15G2R1, IIaA16G3R1, IIdA17G1, and IIdA19G1 have been detected in both lambs and goat kids [33,45,78]. In Gran Canaria, the *GP60* marker revealed that all *C. parvum* isolates found in the sheep and goat farms included in this study belonged to the IId family. However, the genetic heterogeneity was low, as only two distinct subtypes were detected: IIdA16G1 and IIdA23G1. Similarly, subtype IId of *C. parvum* has been reported in small ruminants in various countries, including Algeria [79], Korea [76], Poland [72], and Greece [38], although, in all these studies, subtypes belonging to the IIa family were also observed. Furthermore, the IIdA16G1 and IIdA23G1 subtypes identified in this study have previously been described regarding cattle cryptosporidiosis in Gran Canaria [46], with IIdA23G1 also being the most frequently detected subtype in calves. Many sheep and goat farms in the region follow traditional rearing practices and use shared facilities with other livestock species, such as cattle, which may facilitate inter-species transmission. Notably, since only the IId family was detected in both small and large ruminants sampled in Gran Canaria, it cannot be ruled out that this pattern results from the geographical isolation of *Cryptosporidium* in the archipelago.

The phylogenetic analysis of the *SSU rRNA* gene showed that the sequences of the different species identified in this study clustered with their homologue’s counterparts, often being identical to those available in GenBank. However, the sequence of *C. parvum* (dRP182) in small ruminants reported here did not cluster with a previously identified *C. parvum* sequence from cattle (PP177444) in Gran Canaria [46]. This finding suggests phylogenetic differences between *C. parvum* sequences from large and small ruminants in the Canary Islands. Regarding the phylogenetic analysis of *GP60*, the two subtypes identified in this study not only clustered with previously published sequences in GenBank but also with cattle-derived sequences of the same subtype reported in Gran Canaria (PP333105, PP333107) [46].

Farmers’ knowledge of the aetiological agents causing diarrhoea in lambs and goat kids was analysed by conducting surveys, which also provided additional information on the management systems or hygiene measures applied at the farms. Limitations such as the low number of farms used in the study or the low incidence of infected animals made it difficult to establish an association between these data and the PCR *SSU rRNA* results to detect factors that could favour cryptosporidiosis. Nevertheless, some associations, albeit not statistically significant, could be identified and are discussed below.

An analysis of the survey data revealed an association in kids and goats between a larger farm size and higher positivity rates, as well as a greater number of infected animals per farm, similar to the results reported in [56]. Additionally, this relationship was observed in goat kids and lambs raised under intensive systems compared to extensive ones and in goat kids on farms where no grazing was practiced. These associations suggest that the transmission of *Cryptosporidium* may be enhanced under conditions of increased animal overcrowding. This is not only due to greater contact between animals but also because of the resistance of oocysts shed by infected animals in their faeces, which contaminate the environment, materials, food, and water [80,81]. Regarding other management practices, a higher positivity rate was observed in goat kids on farms with multiple pens for flock separation and in farms with artificial lactation areas, where all the goat kids were housed together in the same space. These results align with previous studies that consider age a risk factor for cryptosporidiosis due to the underdevelopment of the intestinal immune system, particularly in animals less than one month old [82,83].

Regarding the hygienic–sanitary measures, no correlation was observed between the frequency of pen cleaning or manure removal and the *Cryptosporidium* positivity rate of farms. This may be attributed to the high resistance of *Cryptosporidium* oocysts to commonly used disinfectants [84,85]. However, farms that had never conducted parasite testing or vaccinated against only a single disease exhibited higher positivity in goats. This suggests that, overall, these farms implemented fewer biosecurity and control measures.

One of the most significant findings from the surveys was the widespread lack of knowledge among farmers about cryptosporidiosis. Most farmers were entirely unaware of this zoonotic disease, while those who had heard about it placed little or no importance on it. Consequently, no farmers reported outbreaks or clinical signs associated with cryptosporidiosis. Likely for this reason, none of the farms invested in specific treatments against *Cryptosporidium*, which could ultimately result in severe economic losses due to mortality, stunted growth, and increased veterinary costs [14]. Nevertheless, compared to a previous similar study conducted on cattle farms in Gran Canaria, *Cryptosporidium* appears to be of lesser concern in small ruminant farms. This aligns with the relatively low prevalence observed in both young and adult sheep and goats.

## 5. Conclusions

Despite the low incidence, this study confirms the presence of the most important *Cryptosporidium* species in sheep and goats on the island of Gran Canaria, Spain. The role of adult animals as reservoirs for various *Cryptosporidium* species was established through PCR detection, which proved significantly more sensitive than microscopic observation using Kinyoun staining. Furthermore, both detected subtypes of *C. parvum* belonged to the IId family, consistent with a previous study on cattle in the Canary Islands. Additionally, the questionnaire results revealed that most farmers were unaware of the disease, and even those who had some knowledge of it did not consider it important. These findings highlight the urgent need to raise the awareness of this zoonosis and to identify key risk factors for its proper prevention and control. To the best of the authors’ knowledge, this is the first published report describing *Cryptosporidium* species and subtypes in sheep and goat farms on the island of Gran Canaria, Spain.

## Figures and Tables

**Table 1:** Primers used for the different loci and predicted fragment size ranges of PCR-amplified products.

Locus	Primer	Primer Sequence	Fragment Size Range (bp)	Reference
*SSU rRNA*	F1	5′-GGAAGGGTTGTATTTATTAGATAAAG-3′	386–399	[48]
	R1	5′-AAGGAGTAAGGAACAACCTCCA-3′		
	F2	5′-AATTGGAGGGCAAGTCTGGT-3′		
	R2	5′-AACATCCTTGGCAAATGCTT-3′		
*GP60*	F	5′-CCAGCCGTTCCACTCAGA-3′	333–366	[48]
	R	5′-GGTACCTTCTCCGAACCACA-3′		

**Table 2:** Prevalence of *Cryptosporidium* species found with *SSU rRNA* gene marker and families and subtypes of *C. parvum* identified with *GP60* gene marker in lambs, goat kids, sheep, and goat faecal samples in Gran Canaria.

Locus	Nomenclature	GenBank Accession Number	Lambs		Sheep		Goat Kids		Goats	
			Nº of Isolates (n = 84)	Nº of Farms (n = 15)	Nº of Isolates (n = 86)	Nº of Farms (n = 15)	Nº of Isolates (n = 120)	Nº of Farms (n = 15)	Nº of Isolates (n = 120)	Nº of Farms (n = 15)
*SSU rRNA* gene	*C. xiaoi*	PQ345453	2	1	1	1	15	6	1	1
	*C. parvum*	PQ345455	2	2	1	1	13	5	0	0
	*C. ubiquitum*	PQ345467	3	2	4	1	0	0	2	1
*GP60* gene	*C. parvum* IIdA16G1	PQ363713	0	0	0	0	1	1	0	0
	*C. parvum* IIdA23G1	PQ363714	0	0	0	0	6	1	0	0

## Data Availability

The original contributions presented in this study are included in the article. Further inquiries can be directed to the corresponding author.
